# Dynamics of Feline Coronavirus and FIP: A Compartmental Modeling Approach

**DOI:** 10.1155/2023/2721907

**Published:** 2023-11-17

**Authors:** Ayse Peker Dobie, Alper Bayrakal, Mehmet Erman Or, Ayse Humeyra Bilge

**Affiliations:** ^1^Department of Mathematics, Faculty of Science and Letters, Istanbul Technical University, Istanbul, Türkiye; ^2^Department of Internal Medicine, Faculty of Veterinary Medicine, Istanbul University, Istanbul, Türkiye; ^3^Department of Industrial Engineering, Faculty of Engineering and Natural Sciences, Kadir Has University, Istanbul, Türkiye

## Abstract

The investigation of infectious agents invading human and nonhuman populations represents a rich research domain within the framework of mathematical biology, captivating the interest of scientists across various disciplines. In this work, we examine the endemic equilibrium of feline coronavirus and feline infectious peritonitis by using a modified susceptible-infected-susceptible epidemiological model. We incorporate the concept of mutations from FCoV to FIP to enrich our analysis. We establish that the model, when subjected to reasonable parameter ranges, supports an endemic equilibrium wherein the FCoV group dominates. To demonstrate the stability of the equilibria under typical parameters and initial conditions, we employ the model SCF presented by Dobie in 2022 (Dobie, 2022). We ascertain that the equilibrium values reside within the interior domains of stability. Additionally, we displayed perturbed solutions to enhance our understanding. Remarkably, our findings align qualitatively with existing literature, which reports the prevalence of seropositivity to FCoV among stray cats (Tekelioglu et al. 2015, Oğuzoğlu et al. 2010, Pratelli 2008, Arshad et al. 2004).

## 1. Introduction

Feline coronavirus (FCoV) is an enveloped, positive-stranded RNA virus belonging to the family Coronaviridae within the order Nidovirales [1, 2]. This highly contagious virus was first discovered by Ward [3] a few years subsequent to the first recognition of feline infectious peritonitis (FIP) in 1963 at the Angell Memorial Animal Hospital in Boston, as documented by Holzworth [4]. FCoV manifests in two serotypes that diverge in their biological behavior, yet their morphological distinctions are indiscernible [5, 6]. Type 1 (FCoV-I) represents the prevailing serotype and is purely feline in origin [7–9], whereas type 2 (FCoV-II) is relatively uncommon and arises through recombination between FCoV-I and canine intestinal coronavirus (CCoV) [10, 11]. Both serotypes of feline coronavirus possess the capability to induce FIP [12].

Due to its high level of contagiousness, FCoV is prevalent, particularly in multicat environments. The primary mode of transmission for FCoV is indirect, occurring through the faecal-oral route via contaminated cat litter objects and shared litter trays among cats that are either persistently or transiently infected [8, 13]. An infected cat typically begins shedding the virus in their faeces within 2-3 days after infection [14]. The duration of virus shedding varies, spanning weeks to months for FCoV-I, while the exact duration for FCoV-II remains unknown, although experimental infections have indicated approximately 2 weeks [8, 13]. FCoV infections often proceed without noticeable symptoms, posing challenges in terms of diagnosis. Most infected cats eventually recover over time, developing temporary immunity and ceasing viral excretion in their faeces [13, 15]. However, there is a possibility of reinfection. Conversely, in some cats, FCoV can persist, turning them into lifelong carriers of the virus. These cats, referred to as healthy carriers, continue to disseminate the virus through their faeces [8, 16].

Within the population of FCoV-infected cats, the virus mutates at a relatively low incidence rate, up to 10 percent, leading to the development of feline infectious peritonitis (FIP), a highly fatal systemic immune-mediated disease [8, 17]. This percentage of mutation occurrence tends to be higher in kittens with immature immune systems [13, 18]. In the case of newborns from infected mothers, the disease typically emerges between 5 and 7 weeks of age when maternally derived antibodies diminish [19]. Following the initial FCoV infection, it may take several months for FIP to manifest, representing a significant infectious cause of mortality among cats worldwide [20–23]. Most fatalities from FIP are observed in cats between 3 and 16 months of age, with occurrences becoming increasingly rare after the age of 5 years [24]. FIP has two clinical forms: effusive (wet) and noneffusive (dry) [24]. Both forms are progressive and ultimately lead to a fatal outcome [20, 25]. The effusive form, which affects body cavities, is more prevalent and exhibits a more rapid progression compared to the noneffusive form, which targets various organs. Given the absence of a cure and the aggressive nature of the disease, euthanasia is often deemed necessary for cats afflicted with FIP.

This article is inspired by recent observations that a significant proportion of stray cats in Turkey exhibits seropositivity for FCoV. Notably, one survey [26] conducted between January 2009 and April 2014 in Istanbul, Turkey, encompassed a total of 169 cats from various backgrounds, including household, shelter, and stray cats, exhibiting symptoms associated with feline viral infections such as fever, weight loss, depression, and dullness. The findings of this study revealed a high prevalence of FCoV infection, with an upward trend in the number of infected cats over time. Seropositivity rates were reported as 31% in 2009, 25% in 2010, 11% in 2011, 31% in 2012 and 2013, and a striking 83% in 2014. In another survey conducted in 2010, a random selection of 53 cats (20 outdoor and 33 indoor) from different cities in Turkey, without clinical signs of feline viral infections, demonstrated widespread FCoV infection, with nearly 70% of the cats exhibiting seropositivity [27]. It is worth mentioning that the seropositivity rates were reported as 66.6% for female cats and 75% for male cats. Furthermore, a similar study conducted in 2008 in southern Italy revealed that out of 120 samples collected from clinically healthy cats predominantly living in multicat environments (with only 19 in single-cat households), 96 samples tested seropositive, accounting for 82% of the sample population [28]. Additionally, a survey conducted in Malaysia in 2009 involved the selection of 24 cats from four different catteries with a history of at least one confirmed or highly suspected FIP-positive case. The study found that all 24 cats included in the survey tested positive for FCoV [29].

Within the framework of compartmental epidemiological models, which serve as valuable tools for assisting animal health policy development and disease prevention and control [30], the aforementioned literature findings indicate the presence of an endemic equilibrium wherein both healthy and infected individuals can coexist. Building upon this premise, our objective is to investigate the endemic equilibrium of FCoV using a compartmental model initially developed by Kermack and McKendrick in 1927 for the mathematical modeling of infectious diseases [31–35].

Since the groundbreaking contributions of Kermack and McKendrick, significant progress has been made in the study of infectious diseases, including both human and animal populations, as well as the modeling of social interactions. These works have involved a wide range of diseases, including vector-borne diseases, sexually transmitted diseases, and even substance abuse. Notably, more sophisticated models have emerged that account for latent periods, age structure, and various control measures such as isolation, quarantine, and vaccination [36–46]. These compartmental models have demonstrated remarkable predictive accuracy when applied to real-life epidemics. Among these models, the susceptible-infectious-susceptible (SIS) model holds particular relevance for capturing the dynamics of FCoV spread. This model divides the population into two distinct groups: susceptible individuals (S), who have not yet been infected and are susceptible to the disease, and infected individuals (I), who are capable of transmitting the disease to susceptible individuals. Importantly, in the SIS model, there is no permanent immunity following recovery from the infection. However, it should be noted that while the SIS model provides a suitable framework for modeling FCoV, it fails to explain its mutation to FIP.

Motivated by the aforementioned considerations, we use a modified version of the compartmental model proposed by Dobie [47], which has several distinguishing features. In contrast to the single infectious group in the conventional SIS model, our model, denoted as SCF, incorporates two distinct infectious groups, namely I_1_ = C and I_2_ = F. Here, C and F represent diseases caused by the ancestor and mutated viruses, respectively, with neither group exhibiting permanent immunity. Within our model, the C and F infectious groups correspond to cats infected with FCoV and FIP, respectively. The SCF model acknowledges the possibility of disease transmission occurring both horizontally and vertically, allowing for transmission from an infected mother to her offspring. Specifically, we assume that all newborns from the C-infected population belong to the same infectious group. This assumption is based on the fact that unless strict control measures are applied, healthy kittens born to infected mothers will inevitably become infected during the postweaning period. Consequently, the net population growth rate in the C-infected group may have either positive or negative values. Conversely, the mutated virus, responsible for the lethal and incurable FIP, causes a negative net population growth rate in the F-infected group. The SCF model further accounts for the fact that only a fraction of the infected population is subject to virus mutation. In our study, we incorporate this aspect by assuming a relatively low mutation rate, approximately one order of magnitude smaller than the recovery rate. This assumption aligns with the observation that the incidence rate of mutation from FCoV to FIP is generally low.

In the absence of mutation to the lethal strain, our model simplifies to an SIS model, referred to as the SCS model in this context. It is well-known that an SIS model with a single nonlethal strain exhibits an endemic equilibrium. Over time, the healthy and infected populations coexist with proportions determined by the recovery rate. However, when a second lethal strain is introduced, the survival of the species becomes uncertain, and extinction becomes a possibility. In light of recent observations indicating a high prevalence of FCoV among stray cats in Turkey, it is important to develop a model that captures this situation by exhibiting an endemic equilibrium where the FCoV subpopulation dominates. Moreover, it is essential for this equilibrium to be achievable with realistic parameter values. Thus, our objective is to investigate the existence of an endemic equilibrium in our model within reasonable parameter ranges, ensuring a substantial proportion of the population survives.

The article is organised as follows. In Section 2, we present the mathematical model and discuss the introduction of a strain with temporary immunity into a population in demographic equilibrium. Specifically, in Section 2.1, we describe the model in which there is no mutation, resulting in the coexistence of healthy and FCoV-infected cats (SCS model). This model is well-known to exhibit an endemic equilibrium. In Section 2.2, we extend the model to consider the mutation of FCoV to FIP. This introduces the SCF model, which incorporates the following features:

- There are two infectious groups, C and F, representing diseases caused by the ancestor and mutated viruses, respectively, with a mutation rate denoted as *θ*
- There is temporary immunity for individuals in the C-infected group recovering at a rate denoted as *η*, while no recovery occurs in the F-infected group
- The mutated virus leads to a lethal and incurable disease, resulting in a negative net population growth rate, denoted as *f*_2_, for the F-infected group
- The net population growth rates, denoted as *f*_0_ and *f*_1_, in the susceptible and C-infected groups, respectively, can be either zero or have either positive or negative values

Next, we discuss the model that incorporates the deadly mutation of the FCoV strain and analyze the endemic equilibria of this model.  Section 3 focuses on investigating specific cases, and we also present perturbations of the nominal parameter values for these cases. Finally, in the concluding section, we provide a discussion of the results.

## 2. Mathematical Model

In this section, we begin by introducing the standard SIS model, which considers a single strain in a population in demographic equilibrium. We provide a detailed description of the model, including the relevant equations and assumptions. Next, we present a simplified version of the model proposed in [47]. This simplified model represents the scenario where a second mutated deadly strain is introduced into the population. We outline the key features of this model, including the equations and assumptions specific to this situation. To investigate the endemic equilibria, we choose a set of nominal parameter values that serve as a baseline for our investigation. By examining the endemic equilibria and their corresponding dynamics, we aim to gain insights into the impact of introducing a deadly mutated strain on the population dynamics.

### 2.1. SCS Model

**Figure 1:**
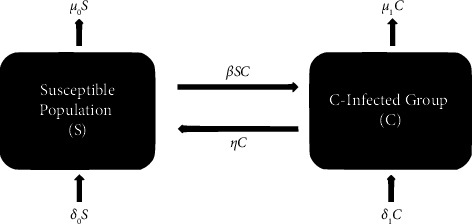
Diagram of the SCS model.

In a population in demographic equilibrium, where the birth and death rates are equal, we can consider the spread of a disease that confers temporary immunity using an SIS model. In this model, individuals can transition between being susceptible to the disease (*S*) and being infected with the disease (*C*). The dynamics of the SIS model can be described by the following set of differential equations:

(1) $$\begin{array}{c} S^{\prime }=-\beta \text{SC}+\eta C+\left(\delta_{0}-\mu_{0}\right)S,\\ C^{\prime }=\beta \text{SC}-\eta C+\left(\delta_{1}-\mu_{1}\right)C, \end{array}$$

where *β* is the infection rate, *η* is the recovery rate, and *δ*_*i*_ and *μ*_*i*_ are the birth and death rates in the group of susceptible and infected individuals for *i*=0 and *i*=1, respectively. For the model defined by (1), interrelations among the compartments are illustrated in Figure 1.

In the case of demographic equilibrium, the birth rates and death rates in each group are equal.

(2) $$\begin{array}{c} \delta_{0}=\mu_{0},\delta_{1}=\mu_{1}, \end{array}$$

meaning that the rates of new individuals entering the population through birth and leaving the population through death are balanced. In this case, the total number of individuals in the population remains constant. In this scenario, it is indeed possible to reach an endemic equilibrium, where both the susceptible (*S*) and infected (*C*) compartments are in demographic equilibrium.

Since the total number of individuals in the population is constant, it is possible to normalize the total population to 1. Thus, in the endemic equilibrium, the final values of *S* and *C* are

(3) $$\begin{array}{c} S_{f}=\left(\frac{\eta }{\beta }\right),C_{f}=\left(\frac{1-\eta }{\beta }\right). \end{array}$$

**Figure 2:**
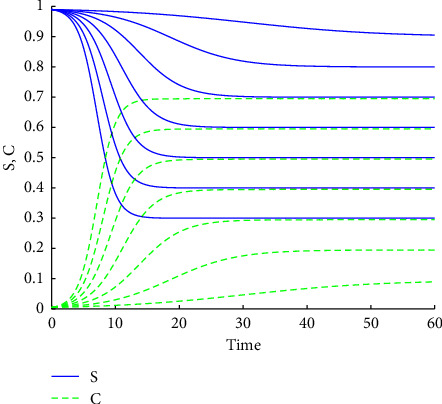
*S*(*t*) and *C*(*t*) for *η*/*β* ranging from 0.3 to 0.9.

The curves of *S*(*t*) and *C*(*t*) for *η*/*β* ranging from 0.3 to 0.9 are demonstrated in Figure 2.

### 2.2. Mutation of a Strain and Endemic Equilibria for a Special Case

**Figure 3:**
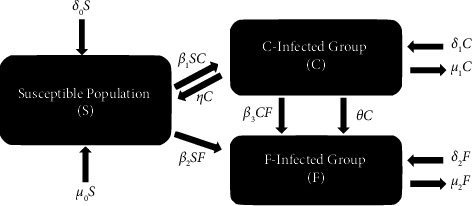
Diagram of the SCF model.

We now consider the case where a second, deadly strain is introduced in the population by a mutation of the first strain. Then, by modifying the SIS model to incorporate this new strain, we obtain the following system governing the spread of these diseases:

(4) $$\begin{array}{c} S^{\prime }=-\beta_{1}\text{SC}-\beta_{2}\text{SF}+\eta C+\left(\delta_{0}-\mu_{0}\right)S,\\ C^{\prime }=\beta_{1}\text{SC}-\beta_{3}\text{CF}-\eta C-\theta C+\left(\delta_{1}-\mu_{1}\right)C,\\ F^{\prime }=\beta_{2}\text{SF}+\beta_{3}\text{CF}+\theta C+\left(\delta_{2}-\mu_{2}\right)F, \end{array}$$

where *θ* is the mutation rate, *β*_2_ and *β*_3_ are the infection rates between *S* − *F* and *C* − *F*, respectively, and *δ*_2_ and *μ*_2_ are the birth and death rates of *F*. Interrelations among the compartments in (4) are illustrated in Figure 3.

To simplify the notation, let us introduce the following new variables:

(5) $$\begin{array}{c} f_{0}=\delta_{0}-\mu_{0},f_{1}=\delta_{1}-\mu_{1},f_{2}=\delta_{2}-\mu_{2}. \end{array}$$

As the groups of individuals are in demographic equilibrium, *f*_0_ and *f*_1_ should be close to zero. On the other hand, as the second strain is deadly, *f*_2_ is negative and its absolute value, and |*f*_2_| is large compared to |*f*_0_| and |*f*_1_|.

The rate of change of the total population (*N*) is

(6) $$\begin{array}{c} N^{\prime }=\left(S+C+F\right)\prime =f_{0}S+f_{1}C-\left|f_{2}\right|F. \end{array}$$

Let us first assume that *f*_0_=*f*_1_=0, but *f*_2_ ≠ 0. If the mutation rate (*θ*) is nonzero, then the equations in (4) together with (6) yield *C*_*f*_=*F*_*f*_=0. Hence, an endemic equilibrium can not be obtained. Therefore, we will proceed with nonzero *f*_0_ and *f*_1_.

To explain the dynamics of the system, we adopt specific parameter values that show the characteristic behavior of the solution curves. In our illustration, we assume equal transmission rates, and by normalizing time (*t*), we choose *β*_1_=*β*_2_=*β*_3_=1. Considering the similar demographic properties of groups *S* and *C*, we make the assumption that *f*_0_=*f*_1_. Additionally, due to the high mortality rate in group *F*, we choose |*f*_2_|=10*f*_1_. Considering the low mutation rate, we set *θ*=0.1*η*. By normalizing the final values as *S*_*f*_+*C*_*f*_+*F*_*f*_=1, linear equations determine the final values of *S*, *C*, and *F*; however, there exists a nonlinear relationship between the parameters. In the case where *β*_2_=*β*_3_, this relationship reduces to a linear equation for *f*_1_. Introducing the scaling *η*=*aθ* and |*f*_2_|=*qf*_1_, we can express *f*_1_ as a rational function of *θ*:

(7) $$\begin{array}{c} f_{1}=\frac{\theta^{2}\left(a+1\right){\left(q+1\right)}^{2}+\theta \left(\beta_{2}-q\right)\left(q+1\right)-\beta_{2}q}{\theta {\left(q+1\right)}^{2}-q\left(q+1\right)}. \end{array}$$

Finally, for *a*=*q*=10 and *β*_2_=1, we obtain

(8) $$\begin{array}{c} S_{f}=\eta +\theta -f_{1}+\frac{1}{11},C_{f}=-\eta -\theta +f_{1}+\frac{9}{11},F_{f}=\frac{1}{11},\\ f_{1}=\frac{1331\theta^{2}-99\theta -10}{121\theta -110}. \end{array}$$

**Figure 4:**
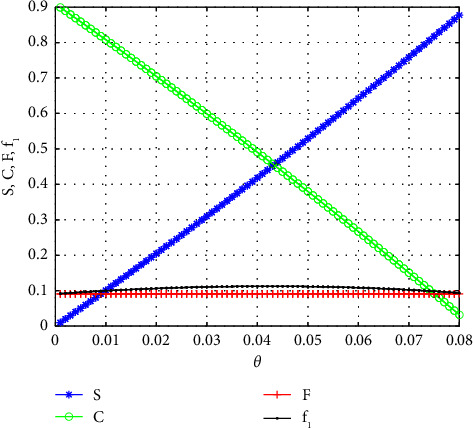
*S*, *C*, *F*, and *f*_1_ as functions of *θ*, for *β*_1_=*β*_2_=*β*_3_=1, *f*_0_=*f*_1_, |*f*_2_|=10|*f*_1_|, *η*=10*θ*.

For these values, the graphs of *S*, *C*, *F*, and *f*_1_ versus *θ* are shown in Figure 4. It has also been checked that these quantities are positive for *θ* < 0.08.

**Figure 5:**
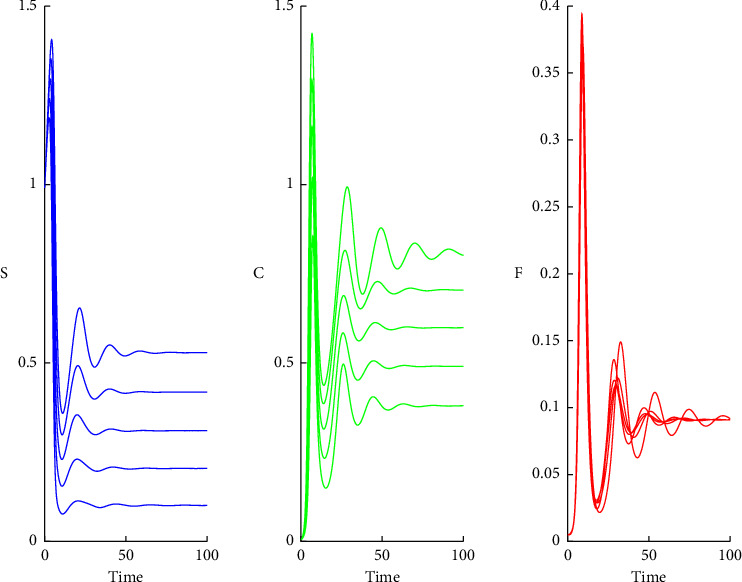
Solution curves for *S*, *C*, and *F* for *θ*=0.01 to 0.05, *β*_1_=*β*_2_=*β*_3_=1, *f*_0_=*f*_1_, |*f*_2_|=10|*f*_1_| and *η*=10*θ*.

Solution curves for *θ* ranging from 0.01 to 0.05 are shown in Figure 5. For *θ* > 0.05, solutions become too oscillatory in the initial period, and these cases are omitted for the clarity of the presentation.

### 2.3. Existence of Endemic Equilibria

In the study proposed by Dobie [47], the parameter ranges for the presence of stable endemic equilibria were extensively investigated. However, in our current work, our focus is not on analyzing the entire spectrum of stable endemic equilibria. Instead, we aim to concentrate on identifying and examining stable equilibria that accurately represent both demographic and epidemiological aspects of the system, thus ensuring their realism and relevance in practical contexts.

In our search for an endemic equilibrium characterized by a nonzero *C*_*f*_ value, we adopt the normalization approach, where the final values are scaled to a total population size of 1. To find the endemic equilibrium, we consider the long-term behavior of the system as *t* approaches infinity, where the right-hand sides of equation (4) become zero. Specifically, their sum equates to zero, yielding the relationship *f*_0_*S*_*f*_+*f*_1_*C*_*f*_ − |*f*_2_|*F*_*f*_=0. By rearranging this equation, we can express *F*_*f*_ in terms of *S*_*f*_ and *C*_*f*_ as follows:

(9) $$\begin{array}{c} F_{f}=\frac{f_{0}}{\left|f_{2}\right|}S_{f}+\frac{f_{1}}{\left|f_{2}\right|}C_{f}. \end{array}$$

Next, we substitute the expression for *F*_*f*_ from the second line of equation (4) and divide the resulting equation by *C*_*f*_, assuming that *C*_*f*_ is nonzero (i.e., *C*′/*C*=0). We then solve for *C*_*f*_ from this equation as

(10) $$\begin{array}{c} C_{f}=\frac{1}{\beta_{3}f_{1}}\left[\left(\beta_{1}f_{2}-\beta_{3}f_{0}\right)S_{f}-\left(\eta +\theta -f_{1}\right)\left|f_{2}\right|\right]. \end{array}$$

The expression for *S*′=0 is quadratic in *S*_*f*_. To simplify the equation, we multiply it by *β*_3_*f*_1_, resulting in the following expression:

(11) $$\begin{array}{c} 0=-S_{f}^{2}\beta_{1}\left(\beta_{1}\left|f_{2}\right|+\beta_{2}f_{1}-\beta_{3}f_{0}\right)+S\left(\eta +\theta -f_{1}\right)\left(\beta_{1}\left|f_{2}\right|+\beta_{2}f_{1}-\beta_{3}f_{0}\right)\\ +S\left(\beta_{1}\left|f_{2}\right|\eta +\beta_{3}f_{0}\theta \right)-\eta \left|f_{2}\right|\left(\eta +\theta -f_{1}\right). \end{array}$$

Instead of solving *S*_*f*_ from the quadratic expression, we solve it from the normalization condition *S*_*f*_+*C*_*f*_+*F*_*f*_=1 to obtain *S*_*f*_ as follows:

(12) $$\begin{array}{c} S_{f}=\frac{\left(f_{1}+f_{2}\right)\left(\eta +\theta -f_{1}\right)+\beta_{3}f_{1}}{-f_{0}\beta_{3}+f_{1}\left(\beta_{1}+\beta_{3}\right)+\left|f_{2}\right|\beta_{1}}. \end{array}$$

When we substitute the expression for *S*_*f*_ in *C*_*f*_ and *F*_*f*_, and rearrange the equations, we obtain the following expressions:

(13) $$\begin{array}{c} S_{f}=\frac{1}{\Delta }\left[f_{1}\left(\beta_{3}+\kappa \right)+\left|f_{2}\right|\kappa \right],\\ C_{f}=\frac{1}{\Delta }\left[f_{0}\left(-\beta_{3}-\kappa \right)+\left|f_{2}\right|\left(\beta_{1}-\kappa \right)\right],\\ F_{f}=\frac{1}{\Delta }\left[f_{0}\kappa +f_{1}\left(\beta_{1}-\kappa \right)\right], \end{array}$$

where

(14) $$\begin{array}{c} \kappa =\eta +\theta -f_{1},\\ \Delta =-\beta_{3}f_{0}+\left(\beta_{1}+\beta_{3}\right)f_{1}+\beta_{1}\left|f_{2}\right|. \end{array}$$

When we substitute the expression of *S*_*f*_ in *S*′=0, we obtain a relation between *f*_*i*_(*i*=0,1,2), *β*_*i*_(*i*=1,2,3), *η* and *θ*. If we define

(15) $$\begin{array}{c} \phi =\beta_{1}\left|f_{2}\right|+\beta_{2}f_{1}-\beta_{3}f_{0}, \end{array}$$

the equation *S*′=0 gives

(16) $$\begin{array}{c} \left(S_{f}\beta_{1}-\kappa \right)\left(\left|f_{2}\right|\eta -S_{f}\phi \right)+S_{f}\beta_{3}f_{0}\theta =0. \end{array}$$

In order to simplify the presentation of this relation, we define

(17) $$\begin{array}{c} f_{0}=f_{1}+P,\beta_{3}=\beta_{2}+Q. \end{array}$$

Then,

(18) $$\begin{array}{c} S_{f}=\frac{\left(f_{1}+\left|f_{2}\right|\right)\kappa +f_{1}\left(Q+\beta_{2}\right)}{\left(f_{1}+\left|f_{2}\right|\right)\beta_{1}-P\left(Q+\beta_{2}\right)}. \end{array}$$

In a realistic situation where *β*_2_=*β*_3_ and *f*_0_=*f*_1_, we have *P*=*Q*=0. In this case, the expression for *S*_*f*_ is simplified to *S*_*f*_=*κ*/*β*_1_. This specific case will be further discussed and analyzed in detail in the next section.

## 3. Numerical Simulations

To ensure realistic demographic and epidemiological constraints, we scale the parameters by orders of magnitude in comparison to the mutation parameter *θ*. Based on these considerations, we make the following assumptions regarding the ranges of the parameters:

(i) The mutation parameter *θ* serves as the basic reference for determining the magnitudes of the parameters in the system.
(ii) The growth rates of the groups *S* and *C* are assumed to be approximately in line with demographic equilibrium. Thus, it is reasonable to scale |*f*_0_| and |*f*_1_| to be of the same order of magnitude. To achieve this, we introduce a scaling factor *α* and express the scaled growth rates as follows:
  (19) $$\begin{array}{c} f_{0}=\alpha \theta ,f_{1}=\alpha \theta . \end{array}$$
Here, *α* does not necessarily need to be close to 1, but it is important for *f*_0_ and *f*_1_ to be relatively “small” in order to obtain realistic solutions.
(iii) The second strain in the model is considered to be deadly, implying that the birth rate *δ*_2_ associated with it is either zero or very small. Consequently, the growth rate *f*_2_ for this strain is negative. In order to capture the severity of this strain, we assume that |*f*_2_| is greater than |*f*_0_| and |*f*_1_| by approximately an order of magnitude. Specifically, we set
  (20) $$\begin{array}{c} \left|f_{2}\right|=10\left|\alpha \right|\theta . \end{array}$$
(iv) To account for the smallness of the mutation rate *θ* compared to the recovery rate *η* by an order of magnitude, we assume
  (21) $$\begin{array}{c} \eta =10\theta . \end{array}$$
(v) One of the infection rates can be normalized to 1 by the time variable *t*. We will normalize *t* by setting *β*_1_=1.

Since the contact rate of the strain *F* to the other two groups will be exactly the same, it would be realistic to consider the following cases:

- Case 1: Equal infection rates, *β*_1_=*β*_2_=*β*_3_=1
- Case 2: Unequal infection rates, *β*_1_=1, *β*_2_=*β*_3_=0.5
- Case 3: Unequal infection rates, *β*_1_=1, *β*_2_=*β*_3_=2

To determine an operating point for the system, we make the following assumptions. We assume that the growth rates *f*_0_ and *f*_1_ are equal in magnitude, while |*f*_2_| is one order of magnitude larger. This assumption is motivated by the condition *f*_1_(*S*_*f*_+*C*_*f*_) − |*f*_2_|*F*_*f*_=0, which implies that *f*_1_ should be positive. Therefore, we set

(22) $$\begin{array}{c} f_{0}=\alpha \theta ,f_{1}=\alpha \theta ,\left|f_{2}\right|=10\alpha \theta ,\eta =10\theta ,\alpha >0. \end{array}$$

If *β*_2_=*β*_3_, then the expressions of *S*_*f*_, *C*_*f*_, and *F*_*f*_ are independent of *β*_2_, but *α* has *β*_2_ dependency, as follows:

(23) $$\begin{array}{c} \alpha =\frac{10\beta_{2}+\left(110-11\beta_{2}\right)\theta -1331\theta^{2}}{11\theta \left(10-11\theta \right)},\\ S_{f}=\frac{100\theta }{10-11\theta },C_{f}=\frac{10}{11}\frac{10-121\theta }{10-11\theta },F_{f}=\frac{1}{11}. \end{array}$$

**Figure 6:**
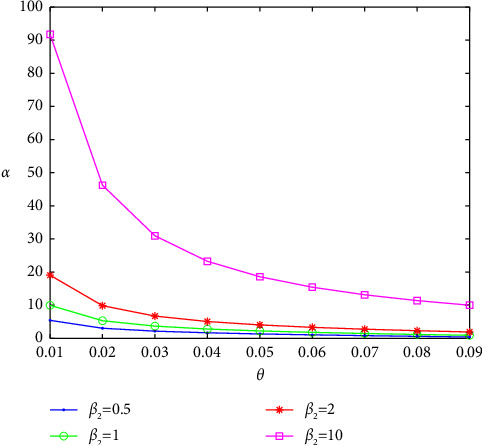
The graphs of *α* versus *θ* for *β*_2_=0.5 (blue curve), *β*_2_=1 (green curve), *β*_2_=2 (red curve), and *β*_2_=10 (magenta curve).

It is easy to see that when *θ* < 10/121, all quantities are positive. Furthermore, the eigenvalues of the Jacobian have negative real parts. The graphs of *α* versus *θ* for *β*_2_=0.5,1,2,10 are shown below in Figure 6.

The regions of stability of endemic equilibria for the SCF model were given in [47], where it has been shown that there are two endemic equilibria characterized by either

(24) $$\begin{array}{c} L<0,K>0,f_{0}<0,f_{1}>0, \end{array}$$

or by the conditions as follows:

(25) $$\begin{array}{c} \left(i\right)\;L>0,f_{0}>0,f_{1}>0,K>\beta_{2}\left(\eta +\theta \right),\beta_{1}\left|f_{2}\right|>\beta_{3}f_{0},\\ \left(ii\right)\;L>0,\;f_{0}>0,\;f_{1}<0,\;K>0,2\theta \left(\beta_{1}\left|f_{2}\right|-\beta_{3}f_{0}\right)-L<0,\\ \left(iii\right)\;L>0,\;f_{0}>0,\;f_{1}<0,\;K>\beta_{2}\left(\eta +\theta \right),\;2\theta \left(\beta_{1}\left|f_{2}\right|-\beta_{3}f_{0}\right)-L>0,\\ \left(iv\right)\;L>0,\;f_{0}>0,\;f_{1}<0,\;K<0,\;2\theta \left(\beta_{1}\left|f_{2}\right|-\beta_{3}f_{0}\right)-L<0,\\ \left(v\right)\;L<0,\;f_{0}>0,\;f_{1}>0,\;K>\beta_{2}\left(\eta +\theta \right),\beta_{1}\left|f_{2}\right|>\beta_{3}f_{0},\\ \left(vi\right)\;L<0,\;f_{0}>0,\;f_{1}<0,\;K>\beta_{2}\left(\eta +\theta \right),\\ \left(vii\right)\;L<0,\;f_{0}<0,\;f_{1}>0,\;K>\beta_{2}\left(\eta +\theta \right),\\ \left(viii\right)\;L<0,\;f_{0}=0,\;f_{1}>0,\;K>0, \end{array}$$

where

(26) $$\begin{array}{c} K=\beta_{1}\left|f_{2}\right|+\beta_{2}f_{1}-\beta_{3}f_{0},J=\eta +\theta -f_{1},\\ L=\text{KJ}-\beta_{1}\left|f_{2}\right|\eta -\beta_{3}f_{0}\theta ,D=L^{2}+4\beta_{3}f_{0}\theta \text{KJ}. \end{array}$$

We have checked that the nominal values of cases 1, 2, and 3 fall in the region (v). We have also checked whether perturbations of these nominal values are still stable. For this, we perturbed all parameters in the system by *ϕ*⟶*ϕ*(1+*ϵξ*) where *ξ* is a normal random variable with zero mean and unit variance, and *ϵ* is a scale parameter. We have run the programs with *N*=1000 replications, and we controlled whether perturbed values of the parameters still lie in the region (v). We have seen that for cases 1 and 2, *ϵ*=1, that is, 100% of perturbations still lie in the same region, but for Case 3, perturbations stay in the stable region only for *ϵ* < 0.05. To illustrate the change in the behavior of the time evolution of the groups in the species, we have obtained solution curves for various values of *β*_2_ and *θ* as shown below.

Next, we present solution curves for perturbations of the parameters.

## 4. Conclusion

Our research in this study focused on explaining the emergence of a new strain within a species. The prevalence of FCoV infection in cats across different locations worldwide serves as a vivid example of this situation [26–29]. Our motivation stemmed from observations concerning the prevalence of FCoV seropositivity among stray cats in Turkey, where almost all of them exhibited positive antibody titers. Complicating matters further, the situation was aggravated by the fact that FCoV has the capacity to mutate into the highly lethal strain known as FIP. To substantiate this observation with convincing evidence, we employed mathematical models with a careful selection of model parameters, which successfully demonstrated the congruence between the observed scenario and the theoretical outcomes derived from a simplified version of the epidemic model, denoted as SCF, as described in [47]. In this model, the variables C and F represent the populations of cats infected with FCoV and FIP, respectively.

Initially, we analyze an epidemic spread involving two strains, with one being a mutation (F) of the ancestral virus (C). Our focus was on the situation where the mutated strain exhibited high lethality, while the ancestor strain caused a disease with a relatively low mortality rate. It should be noted that in the absence of mutation when the healthy and infected populations attain demographic equilibrium, the final proportions are primarily determined by the recovery rate of group C, as detailed in Section 2.1. In essence, if the recovery rate is substantial, the susceptible population dominates the final state, whereas, if it is relatively low, the final state is dominated by group C.

Subsequently, in Section 2.2, we introduced a rare mutation leading to the emergence of a new strain (F) associated with a lethal disease. When the mutation rate from C to F was significantly lower than the recovery rate of C, the essential characteristics of the model remained largely unaltered, and the SCF model could be regarded as a modification of the SCS model. As the mutated strain proved to be highly fatal, we assumed that disease-related fatalities caused by the mutated virus surpassed the net demographic growth rates of the other two groups.

A higher death rate within the F-infected population corresponds to shorter infectious periods for this group. The parameter *R*_0_ that measures the severity of the disease is proportional to the product of the infectivity of the virus and the duration of the infectious period. Thus, shorter infectious periods lead to lower values of *R*_0_. We recall that the final value of individuals affected by the disease is determined solely by *R*_0_; hence, if the disease ends with fatalities in a short term, the survival probability of the species increases.

**Figure 7:**
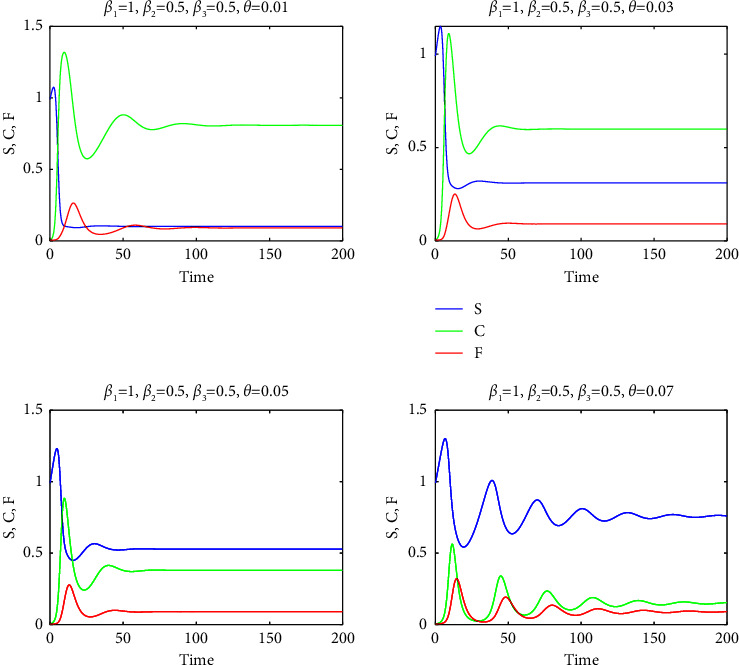
The graphs of *S* (blue curves), *C* (green curves), and *F* (red curves) as a function of *t* for *β*_2_=*β*_3_=0.5 and for *θ*=0.01,0.03,0.05,0.07.

**Figure 8:**
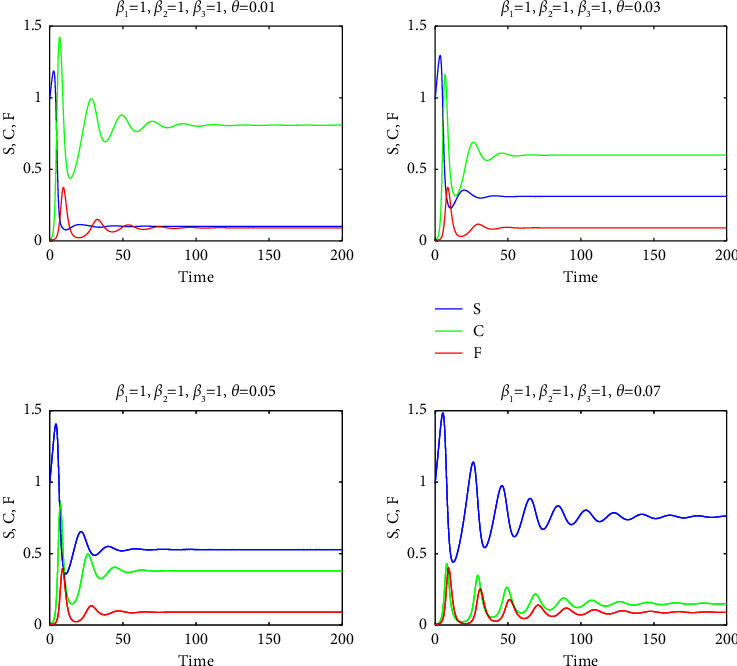
The graphs of *S* (blue curves), *C* (green curves), and *F* (red curves) as a function of *t* for *β*_2_=*β*_3_=1 and for *θ*=0.01,0.03,0.05,0.07.

**Figure 9:**
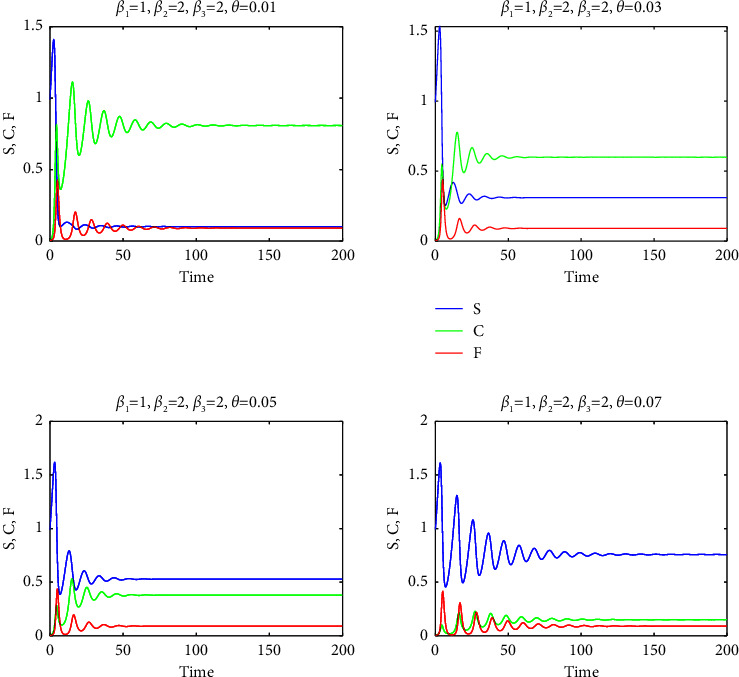
The graphs of *S* (blue curves), *C* (green curves), and *F* (red curves) as functions of *t* for *β*_2_=*β*_3_=2 and for *θ*=0.01,0.03,0.05,0.07.

**Figure 10:**
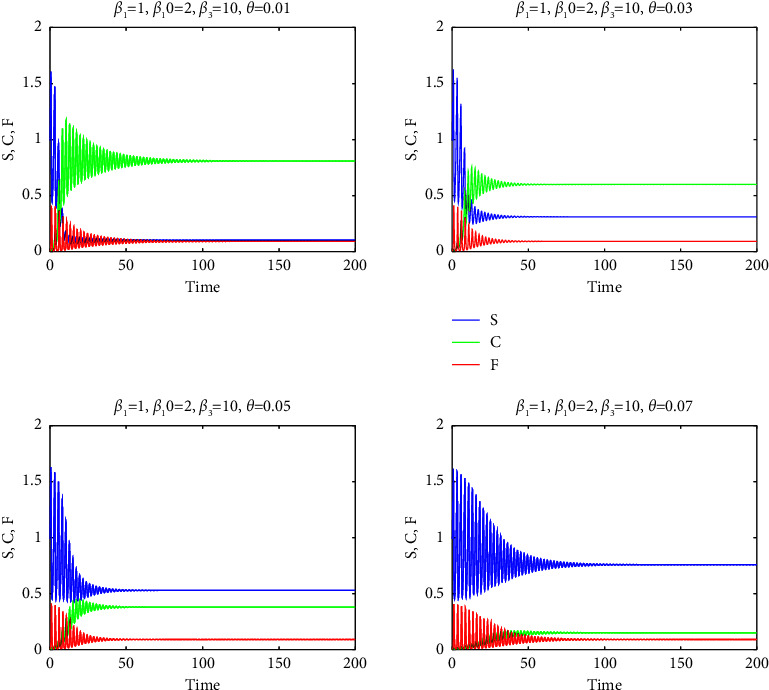
The graphs of *S* (blue curves), *C* (green curves), and *F* (red curves) as functions of *t* for *β*_2_=*β*_3_=10 and for *θ*=0.01,0.03,0.05,0.07.

**Figure 11:**
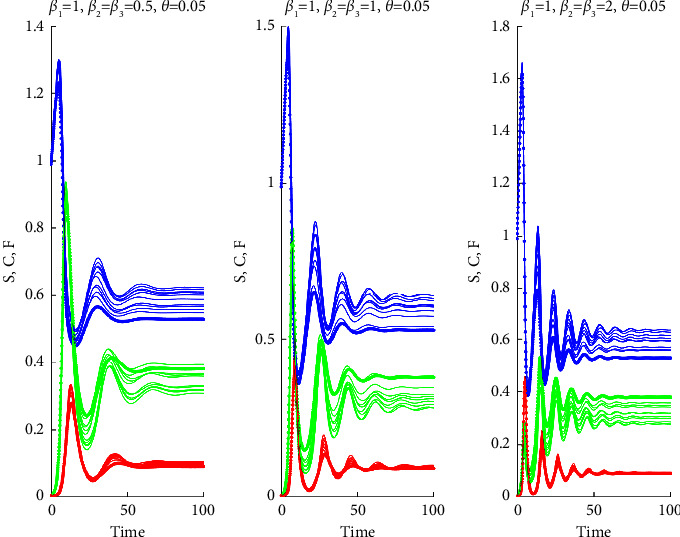
The graphs of *S* (blue curves), *C* (green curves), and *F* (red curves) as a function of *t* for *θ*=0.05 and for *β*_2_=*β*_3_=0.5,1,2.

Consequently, a relatively high mortality rate in the F-infected group, as used in our study, ensured the persistence of substantial populations in groups S and C within the final endemic equilibrium, as evidenced by Figures 7–11. It is important to note that a positive demographic growth rate was also crucial to compensate for the high fatality rates in group F, enabling the existence of an endemic equilibrium. Furthermore, we observed that if the ratio of infection rates (*β*_2_/*β*_1_) was excessively large, the required growth rate for species survival became unrealistic. However, we obtained realistic results for (*β*_2_/*β*_1_) values of 0.5, 1, and 2. Figures 7–9 present solution curves for (*β*_2_/*β*_1_)=*k*, where *k*=1, 0.5, 2, and 10, with *θ*=0.01, 0.03, 0.05, and 0.07. In all cases, as expected, we observed that for lower mutation rates *θ* (and hence, lower recovery rates *η*), the final population was predominantly composed of the FCoV group (C). However, as the mutation rate (and recovery rate) increased, the final population was increasingly dominated by susceptibles. It is worth mentioning that the solutions reached steady states after some oscillations, but this transient behavior appeared to be unrealistic for *k*=10.

Realistic scenarios often confirm deviations in system parameters from their nominal values. We considered these fluctuations around nominal values by running our model with additive random perturbations at the scales discussed in Section 3. Notably, our model demonstrated that the main characteristics of the endemic equilibrium persisted despite these perturbations, as shown in Figure 11, where the qualitative properties of the solutions corresponding to nominal parameter values are preserved. Nevertheless, although not discussed here, there are parameter ranges for which the species may be extinct. The characterization of parameter regions that lead either to extinction or to endemic equilibria is a crucial problem that needs to be addressed in the case of the emergence of an epidemic.

As a result, our model provided compelling evidence for the presence of realistic parameter values that give rise to an endemic equilibrium. Within this equilibrium, the FCoV population asserts its dominance while coexisting with comparatively smaller populations of the other two groups. We should note that the mutation and loss of immunity rates cannot be controlled by human interventions, and lower mutation rates are not always better for the survival of the species. In addition, the parameters *β*_*i*_ that control the spread of the epidemic are proportional to contact rates, and they can be modified by control measures; however, within the parameter ranges used in the present work, the contagion rate has little influence on the final proportions of S, C, and F subgroups. Finally, it is crucial to underscore the pivotal role played by the high mortality rate within the FIP group, as it is the key in establishing an equilibrium that safeguards the survival of the species, even in the presence of a nonlethal infection in the final state. This finding highlights the delicate balance between pathogenicity and survival, underscoring the intricate interplay between different subgroups and the necessity of understanding these dynamics for effective management and conservation strategies.

## Data Availability

No data were used to support this study.
